# Serum biomarkers predictive of cure in Chagas disease patients after nifurtimox treatment

**DOI:** 10.1186/1471-2334-14-302

**Published:** 2014-06-03

**Authors:** Cynthia Santamaria, Eric Chatelain, Yves Jackson, Qianqian Miao, Brian J Ward, François Chappuis, Momar Ndao

**Affiliations:** 1 National Reference Center for Parasitology, Research Institute of the McGill University Health Centre, Department of Medicine, Division of Infectious Diseases, Montreal General Hospital, 1650 Cedar Ave., Room R3-137, Montreal, Quebec H3G 1A4, Canada; 2 Drug for Neglected Diseases initiative (DNDi), Geneva, Switzerland; 3 Division of primary care medicine, Geneva University Hospitals and University of Geneva, Geneva, Switzerland; 4 Division of international and humanitarian medicine, Geneva University Hospitals and University of Geneva, Geneva, Switzerland

**Keywords:** Chagas disease, Biomarkers, Treatment, Nifurtimox, Proteomics

## Abstract

**Background:**

Chagas disease (CD), caused by the protozoan *Trypanosoma cruzi*, remains an important public health issue in many Central and South American countries, as well as non-endemic areas with high rates of immigration from these countries. Existing treatment options for CD are limited and often unsatisfactory. Moreover the lack of post-treatment tests of cure limits the development of new drugs. To address this issue, we sought to identify serum biomarkers following nifurtimox (Nfx) treatment that could be used as an early test of cure and/or markers of a therapeutic response.

**Methods:**

Human sera from Chagas patients pre- and post-treatment with Nfx (n = 37) were compared to samples from healthy subjects (n = 37) using a range of proteomic and immunologic techniques. Biomarker peaks with the best discriminatory power were further characterized.

**Results:**

Using serum samples (n = 111), we validated the presence of five key biomarkers identified in our previous study, namely human apolipoprotein A-I (APOA1) and specific fragments thereof and one fragment of human fibronectin (FN1). In chagasic serum samples all biomarkers except full-length APOA1 were upregulated. These five biomarkers returned to normal in 43% (16/37) of the patients treated with Nfx at three years after treatment.

**Conclusions:**

The normalization of biomarker patterns strongly associated with CD suggests that these markers can be used to identify patients in whom Nfx treatment is successful. We believe that these are the first biomarkers predictive of cure in CD patients.

## Background

Chagas disease (CD) is an important public health concern in Latin America, with an estimated eight million persons chronically infected and a total cost due to loss of productivity of ~$1.2 billion US/year [1] and $24.73 billion in health-care costs [2]. There are at least 41,000 new cases of CD due to vectorial transmission each year [3]. Regardless of the route of transmission (eg: triatomine insects, transfusion, mother-to-child, transplantation, or contaminated foods), the majority of individuals infected by *T. cruzi* remain asymptomatic for life [4]. However, 20-30% will eventually suffer cardiovascular, gastrointestinal, and/or neurological complications [1]. Once exclusively an ‘exotic’ disease, CD has become a burden in many non-endemic countries through migration, blood transfusion, vertical transmission and organ transplantation. Approximately 300,000 chronically-infected individuals are currently thought to be living in the United States [5]. Many European countries, Australia, Japan and Canada are experiencing similar problems. For example, Spain currently has >67,000 CD infected immigrants [6].

Despite the migration of large numbers of chronically-infected CD patients in non-endemic countries, CD remains a neglected disease. Since the introduction of Nfx in 1965 and benznidazole in 1971, the development of new drugs for CD has been negligible [7,8]. Parasitological cure rates for benznidazole or Nfx vary with the disease phase, dose, age, geographical origin and treatment duration [9-12]. There is no consensus regarding optimal treatment duration which varies from 30 days to 90 days depending on the tolerance to the drugs. There is also no reliable test of cure that can be used to monitor the effectiveness of treatment in chronic CD patients [13].

Acute CD phase, which lasts for ~ ten weeks, is relatively easy to diagnose by microscopy, polymerase chain reaction (PCR), hemoculture or/and xenodiagnoses [14]. However, the vast majority of CD cases are not recognized and the disease enters the chronic phase when these tests targeting parasite material in the blood become unreliable due to the low and variable levels of parasitemia. Therefore, negative results using these tests cannot be used to either exclude infection or confirm parasite clearance. During this chronic stage, the diagnostic methods of choice are serologic including indirect immunofluorescence (IFA), indirect hemaglutination (IHA) or enzyme-linked immunosorbent assays (ELISA) [15-18]. Unfortunately, these serologic assays often yield inconclusive results [19] and the Pan American Health Organization (PAHO) now recommends the use of two different tests in parallel [20]. Although these tests can remain positive for decades after treatment, many investigators still rely on them to assess cure [18,21-23]. In the last few years, several studies focusing on biomarkers of cure in chronic human disease have been proposed based on hemostatic parameters [24], natriuretic peptides [25], recombinant antigens [26], and lytic antibodies [27]. None of these approaches is particularly sensitive and new biomarkers to monitor responses to CD treatment are needed.

In recent years, mass spectrometric approaches have seen increased use for a wide range of infectious diseases [28-33]. We have used mass spectrometry to identify biomarkers for *Fasciola hepatica* in sheep [34], *Taenia solium* in pigs [35], and *T. cruzi* in chronically infected patients [36]. The purpose of the current study was to test our candidate *T. cruzi* biomarkers in a cohort of adult patients treated with Nfx and followed up after 3 years [21]. In this work, we not only found the same biomarker pattern in the subjects before treatment but confirmed their disappearance in almost half of the subjects after treatment. Based on these findings, we propose an algorithm to identify patients with chronic CD who have been cured by treatment.

## Methods

### Ethical statement

All of the serum samples used in this study were collected at the Geneva University Hospitals as part of a cohort study in 2008 described by Jackson et al. [37]. The protocol of this study was approved by the Ethical Board for Medical Research of the Geneva University Hospitals (protocol 11–162). Written informed consent was obtained from all participants.

### Samples

Participants diagnosed with chronic CD received treatment with Nfx and were called back for a follow-up (FU) serum sample in 2011 [21]. A total of 37 paired CD samples (pre- and post-Nfx or 2008-CD + and 2011-FU respectively) and 37 samples from matched healthy control (HC) with similar epidemiologic risk were available for study. The seronegative status of the HC was confirmed by two serological tests (i.e. BioMerieux Elisa cruzi® and Biokit Bioelisa Chagas®). Patient characteristics and the details of treatment have been described elsewhere [21]. Written informed consent was obtained from all participants. Sera were frozen (-20°C) within 1 hour of collection and stored in a bio-bank at the HUG. Aliquot for each subject and time-point were sent to the National Reference Center for Parasitology (Montreal, QC) for the proteomics analysis.

ELISA and PCR assays have been described previously [21].

### SELDI Analysis

Serum analysis was carried out as described by Ndao et al. [30,36]. Briefly, 20 μl of each serum sample were denatured and fractionated using 96-well filtration plates in a series of ten minute incubations in buffers with decreasing pH. Serial washes yielded fractions 1–5 (pH 9, 7, 5, 4, and 3 respectively) with a last wash in organic buffer (fraction 6). All six fractions were bound on both CM10 (weak cationic-exchange) and IMAC30 (immobilized metal) ProteinChip arrays (Bio-Rad Laboratories, Hercules, CA) as described [30,36]. Arrays were then air-dried and 1 μl of energy-absorbing matrix (sinapinic acid, Bio-Rad) was added twice to each spot. The surface was allowed to air dry between each application. Arrays were analyzed in a ProteinChip system reader (PCS-4000) using the ProteinChip Data Manager Software version 3.5 (Bio-Rad). Each spot was read at low and high-energy laser intensities. Spectra were calibrated and normalized as previously described [36].

Data was analyzed using ProteinChip Data Manager Software as described in detail previously. *P* values for differences in peak intensities between the three groups (2008-CD+, 2011-FU and HC) for each cluster were generated using the Mann–Whitney U test. Diagnostic performance was assessed by determining the area under the receiver-operating characteristic (ROC) curve for each potential biomarker. The Biomarker Patterns Software version 5.0.2 (Bio-Rad), a classification method, was then used to identify peaks with the greatest contribution to discrimination between groups (HC, 2008-CD + and 2011-FU).

### Cross-validation of SELDI results and biomarker identity confirmation

#### *Neo-epitope antisera against human APOA1 and FN1 fragments*

Rabbit anti-peptide sera were generated against the predicted neo-termini of the 24.7 kDa fragment of APOA1 (PALEDL: amino acids 209–214) and the 28.9 kDa fragment of FN1 (GPFTDV: amino acids 253–258) were conjugated to immunogenic KLH (CanPeptide, Pointe-Claire, QC) and sent to Cocalico Biologicals Inc (Reamstown, PA) for rabbit immunization and antiserum production. These antisera recognized the CD-associated fragments but not the native forms.

#### *Immunoblot confirmation of biomarkers presence in serum samples*

Individual and pooled serum samples from HC and paired 2008-CD + and 2011-FU samples were separated in 4-12% Novex Bis-Tris Midi gradient gels (Life Technologies, Carlsbad, CA) under reducing conditions. Separated proteins were transferred to nitrocellulose membranes by iBlot dry transfer (Life Technologies). Transfer efficiency was assessed by Ponceau Red. Membranes were incubated overnight at 4°C with either the rabbit anti-APOA1(24) serum at 1:500 or the rabbit anti-FN1(28) serum at 1:1,000 dilution followed by incubation with HRP-conjugated anti-rabbit IgG at a 1:100,000 dilution (GE Healthcare Life Sciences, Uppsala, Sweden). The membranes were incubated in SuperSignal West Pico detection (Pierce, Rockford, IL) and exposed to autoradiography.

#### *Validation of SELDI results by ZOOM fractionation*

Pools of HC (n = 4) and 2008-CD + (n = 4) samples were fractionated using the ZOOM IEF Fractionator (Life Technologies). The pools (20 μl) were solubilized, denatured and alkylated in a final concentration of 1X IEF Denaturant, 1X protease inhibitor cocktail (EMD4 Biosciences, Calbiochem, Gibbstown, NJ), 40 mM DTT, 5 mM EDTA, 10 mM Tris base and ~50% N,N-dimethylacrylamide. The samples were diluted to a final volume of 3.5 ml with 1.1X IEF Denaturant according to the manufacturer’s protocol. Five fractions of varying pH were collected: pH 10-9.1, pH 9.1-7, pH 7-5.4, pH 5.4-4.6, and pH 4.6-3. Based on the SELDI results, ZOOM fractions pH ranges 5.4-10 were most likely to contain the APOA1 and FN1 fragments. Fractions were separated on Mini-PROTEAN 4-15% TGX gradient gels (Bio-Rad) for immunoblot assessment and in-gel trypsin digestion. Immunoblots were performed as described above.

#### *In-gel trypsin digestion*

The gels were stained with Bio-safe Coomassie stain (Bio-Rad). Gel bands of interest were excised and transferred to Eppendorf tubes for trypsin digestion using proteomics grade recombinant trypsin according to the manufacturer protocol (Roche, Penzberg, Germany). A gel piece from an empty corner of the pre-cast gel was simultaneously processed for use as a blank. The blank sample was used to generate an exclusion list of common contaminating peaks from the gel, the trypsin and the α-cyano-4-hydroxycinnamic acid (CHCA) matrix used during spotting. The samples were desalted using ZipTips C_18_ (Millipore, Billerica, MA) following the manufacturer’s protocol. Samples were eluted with 4 μl matrix solution (10 mg/ml CHCA in 50% ACN and 0.1% TFA) and spotted directly on a 384-well Opti-TOF MALDI plate (AB Sciex, Framingham, MA) in 1 μl aliquots and allowed to air-dry at room temperature.

#### *MALDI MS-MS identification of biomarkers*

MALDI data was acquired using a 4800 Plus MALDI TOF/TOF Analyzer (AB Sciex) with the 4000 Series Explorer software version 3.5.3. The instrument was calibrated using the AB Sciex Mass Standards Kit. Data was first acquired in the MS positive-ion reflector mode at a fixed laser energy of 3200. A reflector interpretation method was created to run the MS-MS 1kv positive acquisition method in batch mode on all spots. The MS-MS was performed at 4200 fixed laser intensity on the 50 strongest precursor peaks discovered during the reflector mode acquisition. The exclusion list of common CHCA, trypsin and gel contaminant peaks was also included as a parameter in the interpretation method to improve for the selection of protein-specific precursor peaks. MS-MS data was analyzed with ProteinPilot software version 4.0.8 (AB Sciex) based on the paragon algorithm. MS-MS data was searched against the most recent *Homo sapiens* protein database from Uniprot (Swiss-Prot/TrEMBL annotated protein sequences).

## Results

### Identification of serum markers from chagasic patients using SELDI

**Figure 1:**
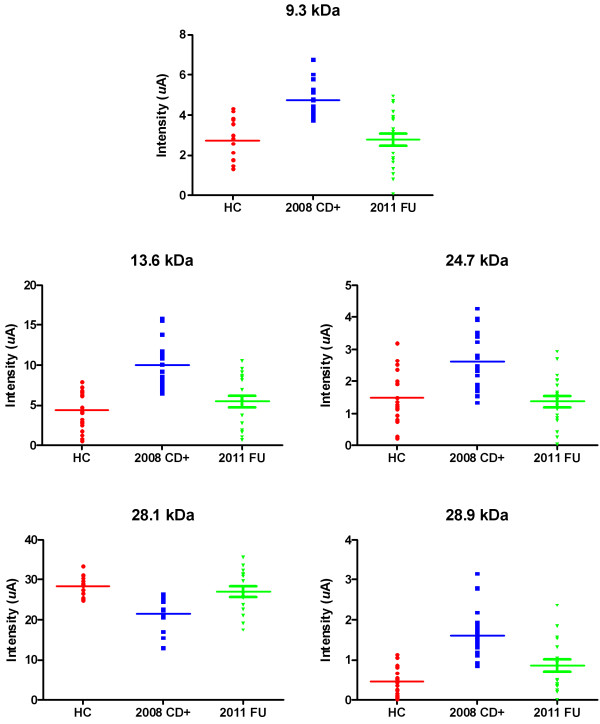
**Cluster plots of selected differentially expressed biomarkers.** Cluster plots demonstrate the expression pattern of selected biomarkers between the different groups (HC = healthy controls; 2008-CD + = chagasic samples; 2011-FU = follow-up samples from patients having received treatment with Nfx). Biomarker expression levels in samples treated with Nfx (2011-FU) all return to the levels observed in healthy controls (HC). All biomarkers, except for one, are upregulated during disease (2008-CD+). The downregulated biomarker was previously identified as native human APOA1 (28.1 kDa); the 9.3, 13.6 and 24.7 kDa biomarkers were identified as fragments of human APOA1; and the 28.9 kDa biomarker as a fragment of human FN1.

The 111 sera studied yielded a total of 2664 spectra. From the list of 18 candidate biomarkers identified in our previous work [36], 15 were also found in the current study (Table 1). All showed statistically significant differences (p < 0.05) in peak intensity between the 2008-CD + samples and HC samples. All but two (5.4 and 28.1 kDa) were up-regulated in CD patients (2008-CD+/HC fold ratio). When the 2008-CD + and 2011-FU were compared, all but one of these 15 candidate biomarkers (5.4 kDa) demonstrated statistically significant intensity shifts towards the normal values of the HC group. Nine of the 15 biomarkers were no longer significantly different between HC and 2011-FU samples (7.8, 9.3, 10.1, 12.7, 13.6, 15.2, 16.3, 24.7 and 28.1 kDa: p-values all > 0.05), strongly suggesting the ‘normalization’ of these biomarkers after treatment with Nfx (Table 1). The cluster plots (Figure 1) show a visual representation of peak intensity levels in the HC, 2008-CD + and 2011-FU groups for five of the best potential biomarkers. Based upon our previous work, most of these biomarkers are of host origin [36]. In particular, the 9.3, 13.6 and 24.7 kDa peaks were identified as human APOA1 fragments and the 28.1 kDa peak is the mature form of APOA1. Another peak of interest is the 28.9 kDa protein previously identified as a fragment of FN1 [36].

**Table 1:** Validation of previously identified Chagas biomarkers within HUG study samples

***m/z *** **(/1,000)**	**Fractions and chemistries**	**P value (CD + vs HC) AUC for ROC curve (fold CD/HC)**	**P value (FU vs HC) AUC for ROC curve (fold FU/HC)**	**P value (CD + vs FU) AUC for ROC curve (fold CD/FU)**	**Mean signal intensity ± SEM**		
					**HC**	**CD+**	**FU**
					**(n = 37)**	**(n = 37)**	**(n = 37)**
3.7	F1 CM10	0.0001, 1.0 (+4.2)	0.0001, 0.88 (+2.3)	0.0011, 0.82 (+1.8)	1.54 ± 0.11	6.46 ± 0.64	3.60 ± 0.45
4.4	F1 IMAC; F1,5 CM10	0.0001, 1.0 (+3.7)	0.0159, 0.69 (+1.7)	0.0001, 0.91 (+2.2)	7.66 ± 0.53	28.35 ± 1.74	12.84 ± 1.91
5.4a	F1, 3 CM10	0.0001, 1.0 (-0.3)	0.0001, 0.99 (-0.2)	0.2427, 0.65 (+1.2)	3.51 ± 0.31	0.90 ± 0.07	0.75 ± 0.10
6.6	F3,5 CM10	0.0001, 1.0 (+3.4)	0.0001, 0.91 (+2.5)	0.0028, 0.79 (+1.4)	3.65 ± 0.26	12.45 ± 0.62	8.96 ± 0.89
7.8	F1,3 CM10	0.0001, 0.97 (+3.9)	0.3405, 0.55 (+1.2)	0.0005, 0.91 (+3.15)	0.55 ± 0.07	2.14 ± 0.35	0.68 ± 0.12
9.3	F1,4 IMAC; F1,3 CM10	0.0001, 0.92 (+1.7)	0.9456, 0.51 (+1.0)	0.0001, 0.87 (+1.7)	2.74 ± 0.26	4.76 ± 0.24	2.77 ± 0.31
10.1	F1,3,4,6 IMAC; F1 CM10	0.0001, 0.88 (+2.0)	0.3622, 0.56 (-0.9)	0.0001, 0.96 (+2.3)	15.27 ± 1.80	30.48 ± 2.92	13.13 ± 1.42
12.7	F2,5 IMAC; F1,5,6 CM10	0.0003, 0.80 (+2.0)	0.5879, 0.53 (+1.2)	0.0129, 0.73 (+1.7)	2.59 ± 0.41	5.27 ± 0.55	3.02 ± 0.68
13.6	F1 IMAC; F1 CM10	0.0001, 0.96 (+2.3)	0.2220, 0.59 (+1.2)	0.0001, 0.86 (+1.8)	4.43 ± 0.51	10.07 ± 0.62	5.50 ± 0.71
15.2	F4,5 IMAC; F1,3,6 CM10	0.0001, 1.0 (+2.9)	0.6538, 0.51 (+1.1)	0.0001, 0.96 (+2.7)	5.62 ± 0.51	16.46 ± 1.33	6.04 ± 0.75
16.3	F4 IMAC; F3,6 CM10	0.0001, 0.95 (+3.1)	0.4349, 0.58 (+1.2)	0.0001, 0.94 (+2.6)	1.77 ± 0.30	5.48 ± 0.48	2.10 ± 0.28
24.7	F2 IMAC	0.0001, 0.84 (+1.8)	0.6429, 0.51 (-0.9)	0.0001, 0.85 (+1.9)	1.48 ± 0.16	2.61 ± 0.19	1.37 ± 0.18
28.1a	F1,3 CM10	0.0001, 0.96 (-0.8)	0.3598, 0.56 (-1.0)	0.0032, 0.79 (-0.8)	28.43 ± 0.63	21.66 ± 1.07	27.06 ± 1.23
28.9	F1 IMAC	0.0001, 0.98 (+3.5)	0.0182, 0.66 (+1.9)	0.0006, 0.80 (+1.9)	0.46 ± 0.07	1.61 ± 0.13	0.86 ± 0.15
75.4	F1 CM10	0.0001, 1.0 (+3.0)	0.0005, 0.83 (+1.9)	0.0001, 0.96 (+1.6)	1.18 ± 0.15	3.53 ± 0.09	2.25 ± 0.23

a Downregulated protein in CD-positive subjects.

Mass (m/z), mean intensities, and AUC’s for selected differentially expressed peptides/proteins between HC, 2008-CD + and 2011-FU sera samples.

An area under the curve (AUC) of 1 suggests good diagnostic power, while AUC values that approach 0.5 indicate little diagnostic potential. HC: Healthy control; CD: Chagas disease; FU: follow-up.

### Immunoblot confirmation of selected markers in sera

**Figure 2:**
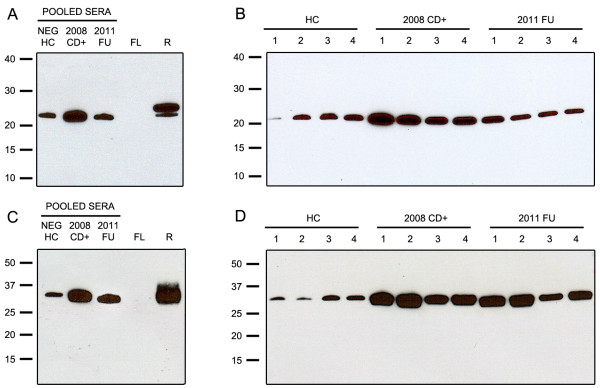
**Immunoblot analysis of sera using rabbit anti-neoepitope antisera.** Immunoblot analysis of sera from healthy controls (HC), 2008 chagasic samples (2008-CD+) and 2011 follow-up samples (2011-FU) probed with specific rabbit neo-epitope antisera. **(A)** Analysis of pooled sera and **(B)** individual sera with anti-APOA1(24) demonstrates the upregulation of the 24.7 kDa APOA1 fragment in 2008-CD + samples and its down-regulation back to healthy controls level in the 2011-FU samples. The specificity of the rabbit anti-APOA1(24) serum for the 24.7 kDa fragment is demonstrated by its inability to detect native full-length APOA1 (~28.1 kDa) (FL) while reacting with a 6xHis recombinant of the 24.7 APOA1 fragment (~27.4 kDa) (R). **(C)** Analysis of pooled and **(D)** individual sera with rabbit anti-FN1(28) serum demonstrates the up-regulation of the 28.9 kDa FN1 fragment in 2008-CD + samples and its down-regulation in the 2011-FU samples almost back to the levels observed in healthy controls. The specificity of the rabbit anti-FN1(28) serum for the 28.9 kDa fragment is demonstrated by its inability to detect native full-length FN1 (~210 and ~220 kDa) (FL) while reacting with a FLAG-6xHis recombinant of the 28.9 FN1 fragment (~35 kDa) (R).

**Figure 3:**
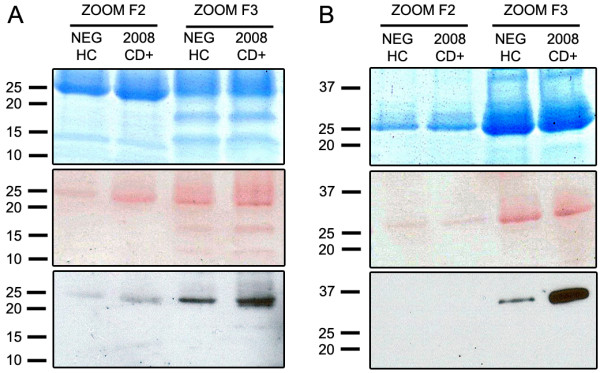
**Immunoblot analysis of ZOOM fractions using specific anti-neoepitope antisera and Coomassie gel used for in-gel trypsin digestion.** Coomassie, Ponceau Red and Immunoblot analysis of pooled sera fractionated by ZOOM IEF Fractionation validate SELDI results. **(A)** ZOOM fractions 2 and 3 probed with anti-APOA1(24) rabbit serum confirms the presence and up-regulation of the 24.7 kDa APOA1 biomarker in 2008-CD + samples vs healthy control (HC) samples especially in fraction 3 (pH range 5.4-7). **(B)** ZOOM fractions 1 and 2 probed with anti-FN1(28) confirms the presence and up-regulation of the 28.9 kDa FN1 biomarker in 2008-CD + vs HC samples in fraction 2 (pH range 7-9.1).

The specificity of the antisera for the targeted fragments is well demonstrated in Figure 2A in which the anti-APOA1(24) detects a single band at ~27 kDa that corresponds to the 6xHis-tagged recombinant APOA1 24.7 kDa fragment (lane R). No band is detected in the lane loaded with the native 28.1 kDa native form of APOA1 (lane FL) purchased from Sigma. Similarly, the anti-FN1(28) detects a single band corresponding to the 6xHis-FLAG recombinant FN1 28.9 kDa fragment (Figure 2C, lane R) but not native FN1 (~210 and ~220 kDa) (Figure 2C, lane FL) was not detected by the anti-FN1(28) (high molecular range data not shown). Staining with Ponceau Red confirmed successful transfer of the full-length proteins to the nitrocellulose (data not shown). Probing individual and pooled samples demonstrated up-regulation of both the 24.7 kDa APOA1 (Figure 2A,B) and 28.9 kDa FN1 (Figure 2C,D) fragments in Chagas patients (2008-CD + lanes) compared to sera from healthy individuals (HC lanes). Band intensities are much reduced three years after Nfx treatment (2011-FU lanes). The immunoblots results were consistent with the patterns observed on both the cluster plots (Figure 1) and the mean signal intensities (Table 1). Immunoblots analysis of ZOOM fractions 2 and 3 confirmed the presence of both the 24.7 kDa APOA1 and the 28.9 kDa FN1 fragments (Figure 3A,B).

### MALDI MS-MS analysis of ZOOM fractions

**Figure 4:**
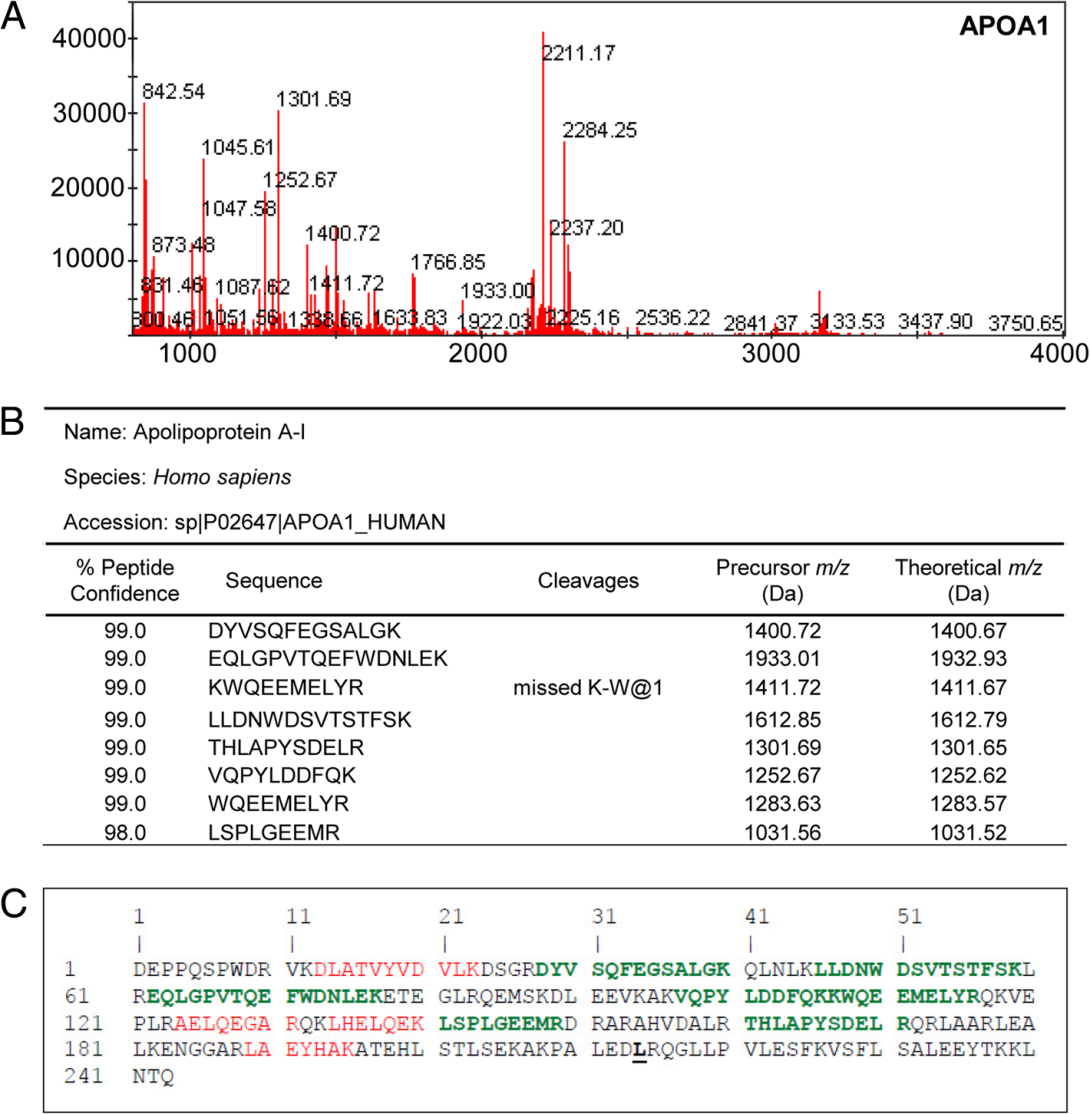
**MALDI identification of human apolipoprotein A-I.** MALDI MS-MS data confirms the identification of the 24.7 kDa biomarker to be a fragment of human APOA1. **(A)** MS Reflector Positive spectrum obtained from the in-gel trypsin digestion of the ~25 kDa band cut out from fraction 3 shows the precursor peaks generated by trypsin. **(B)** ProteinPilot MS-MS data shows that 8 peptides were sequenced and matched to human APOA1 with more than 95% confidence. **(C)** MS-MS sequence coverage. Peptides sequenced with more than 95% confidence (green) or less 95% confidence (red) together covered 47.7% of the full-length APOA1 human protein sequence.

**Figure 5:**
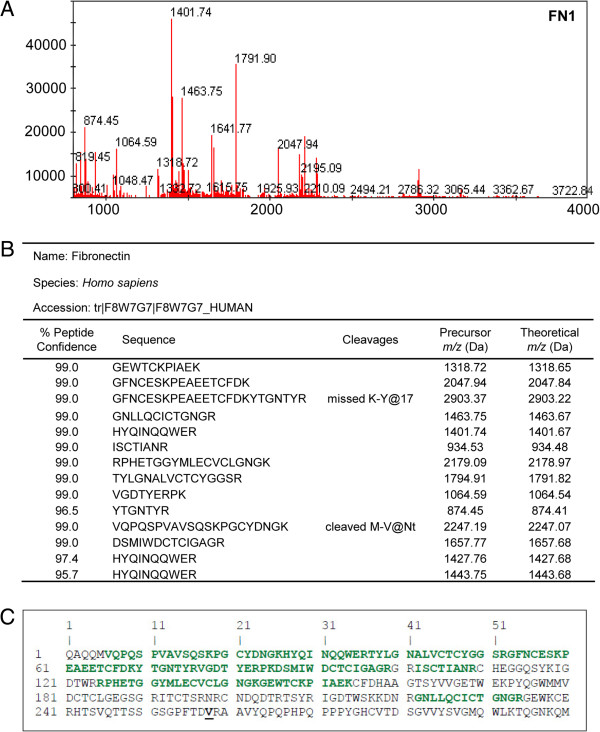
**MALDI identification of human fibronectin.** MALDI MS-MS data confirms the identification of the 28.9 kDa biomarker to be a fragment of human FN1. **(A)** MS Reflector Positive spectrum obtained from the in-gel trypsin digestion of the ~30 kDa band cut out from fraction 2 shows the precursor peaks generated by trypsin. **(B)** ProteinPilot MS-MS data shows that 14 peptides were sequenced and matched to human FN1 with more than 95% confidence. **(C)** MS-MS sequence coverage. Peptides sequenced with more than 95% confidence (green) covered 48.3% of the amino-terminal domain of the FN1 human protein sequence.

Aliquots of ZOOM fractions 2 and 3 were separated by gel electrophoresis and stained (Figure 3A,B). Gel bands at ~25 kDa and ~29 kDa were excised and subjected to in-gel trypsin digestion. The 50 peaks (Figures 4A and 5B) with the highest intensity were subjected to MS-MS sequencing which confirmed the presence of human APOA1 (Uniprot accession#: APOA1_HUMAN) in ZOOM fraction 3 with eight peptides covering 47.7% of the mature protein (Figure 4B,C). The leucine residue at position 214 (highlighted in black bold, Figure 4C) is where we believe mature APOA1 is cleaved by cruzipain to give rise to the 24.7 kDa APOA1 fragment [36]. Since no precursor peptide was identified and sequenced beyond this leucine residue, these data are consistent with the immunoblot results suggesting the presence of the 24.7 kDa APOA1 fragment rather than the full-length protein in ZOOM fraction 3. Similarly human FN1 (Uniprot accession#: F8W7G7_HUMAN) was identified from MS-MS data in ZOOM fraction 2 with >14 peptides covering 48.3% of the N-terminal domain of FN1 (Figure 5B,C). In this case, cruzipain is presumed to cleave full-length FN1 at the valine residue in position 258 (highlighted in black bold, Figure 5C). Again no precursor peptide was identified and sequenced beyond this valine residue.

### Discrimination between groups using the biomarker pattern software

**Figure 6:**
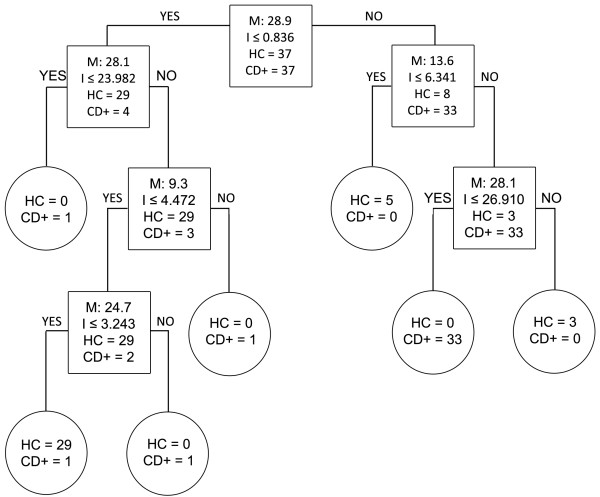
**Biomarker patterns software based on CART analysis used to generate candidate diagnostic algorithms between the HC and 2008-CD + groups.** Biomarker patterns software based on CART (Classification and Regression Tree) analysis was used to generate candidate diagnostic algorithms. Decision tree classifications using healthy controls (HC) vs 2008 chagasic samples (CD+) are shown. Biomarkers establishing the splitting rules are 9.3, 13.6, 24.7, 28.1, 28.9 kDa. Cases that follow the rule are placed in the left daughter node (YES), and samples that do not follow the rule are placed in the right daughter node (NO).

When healthy controls and 2008-CD + samples were compared, the best discriminatory peaks identified by the BPS were 28.9, 28.1, 13.6, 9.3 and 24.7 kDa (Figure 6). These biomarkers corresponded to the 28. 9 kDa FN1 fragment, full-length APOA1 and three of the APOA1 fragments respectively. Taking this into account and assuming that the pattern of biomarkers is strong enough to discriminate between a Chagas sample and a healthy sample, a patient could be considered cured when the identified sera markers have the same profile as that found in healthy volunteers i.e. FN1 fragment (28.9 kDa) and all APOA1 truncations (9.3, 13.6 and 24.7 kDa) are down-regulated and full-length APOA1 (28.1 kDa) is up-regulated. We therefore applied the same algorithm to classify the 2011-FU samples according to the algorithm used to discriminate between healthy controls and Chagas patients in order to define whether a patient can be classified as cured or not.

Table 2 summarizes the patterns of selected biomarkers in follow-up samples from patients treated with Nfx. As described previously, all 37 subjects tested positive by ELISA, making it difficult to draw any conclusion about treatment efficacy [21]. One patient treated for 60 days with Nfx was found to be still Chagas positive as assessed by real-time PCR [21]. The biomarker pattern found for this patient matches that of a Chagas patient (three APOA1 and one FN1 fragments up-regulated and full-length APOA1 down-regulated). Similar patterns should identify treated but not cured patients. Among the patients that received Nfx treatment for less than 40 days, nine out of ten could be classified as Chagas patients, eight of those displaying exactly the same pattern as the only patient positive by real-time PCR. Interestingly, one patient that followed the treatment for 21 days was classified as cured. Of the full cohort of 37 patients, the overall cure rate was 43.2%, with 15 of the 27 patients (55.6%) who received treatment for 47 to 60 days (25 of whom completed the full 60 days) classified as healthy therefore cured, and ten as Chagas patients not cured.

**Table 2:** Expression patterns of selected biomarkers in 2011 follow-up samples compared to 2008 Chagasic patients treated with nifurtimox (Upward arrow: up-regulated; Downward arrow: down-regulated)

**Nfx days**	**Sample Lab ID**	**9.3 kDa**	**13.6 kDa**	**24.7 kDa**	**28.1 kDa**	**28.9 kDa**	**2008 ELISA OD**	**2011 ELISA OD**	**2011 ELISA**	**2011 PCR**	**2011 RT-PCR**	**Cured (C) Not Cured (NC)**
60	54	↓	↓	↓	↑	↓	0.62	0.62	+	-	-	C
60	56	↓	↓	↓	↑	↓	2.53	2.02	+	-	-	C
60	57	↓	↓	↓	↑	↓	0.41	0.50	+	-	-	C
60	59	↓	↑	↓	↑	↓	2.37	2.17	+	-	-	C
60	60	↑	↑	↑	↓	↑	2.80	2.57	+	-	-	NC
60	62	↓	↓	↑	↑	↓	2.55	2.11	+	-	-	NC
60	65	↓	↓	↑	↑	↓	0.79	0.61	+	-	-	NC
60	68	↓	↑	↓	↓	↓	1.88	1.53	+	-	-	NC
60	69	↓	↓	↑	↑	↓	1.84	1.75	+	-	-	NC
60	70	↓	↑	↑	↑	↓	1.11	1.23	+	-	-	NC
60	71	↑	↑	↑	↓	↑	1.64	1.19	+	-	+	NC
60	72	↓	↓	↓	↑	↓	1.47	1.67	+	-	-	C
60	77	↓	↑	↓	↑	↓	2.16	1.58	+	-	-	C
60	84	↓	↑	↓	↑	↓	2.94	2.49	+	-	-	C
60	85	↓	↑	↑	↓	↓	1.76	2.11	+	-	-	NC
60	86	↑	↓	↓	↑	↓	2.18	2.25	+	-	-	NC
60	87	↓	↓	↓	↑	↓	1.95	1.89	+	-	-	C
60	91	↓	↓	↑	↑	↓	2.16	1.83	+	-	-	NC
60	94	↓	↓	↓	↑	↓	2.07	1.93	+	-	-	C
60	97	↓	↓	↑	↑	↓	2.26	1.85	+	-	-	NC
60	99	↓	↑	↓	↑	↓	1.69	1.25	+	-	-	C
60	100	↓	↓	↓	↑	↓	2.17	1.79	+	-	-	C
60	102	↓	↓	↓	↑	↓	2.61	1.31	+	-	-	C
60	103	↑	↓	↑	↓	↑	2.56	2.28	+	-	-	NC
60	104	↓	↓	↓	↑	↓	2.78	1.97	+	-	-	C
58	64	↓	↓	↓	↑	↑	2.55	2.27	+	-	-	C
47	67	↓	↓	↓	↑	↓	1.83	1.90	+	-	-	C
31	58	↑	↑	↑	↓	↑	2.28	1.91	+	-	-	NC
21	80	↓	↑	↓	↑	↓	2.42	1.78	+	-	-	C
21	81	↑	↑	↑	↓	↑	2.16	1.70	+	-	-	NC
21	88	↑	↑	↑	↓	↑	2.02	1.64	+	-	-	NC
19	96	↑	↑	↑	↓	↑	1.99	2.42	+	-	-	NC
16	90	↓	↑	↑	↓	↓	1.44	1.02	+	-	-	NC
14	95	↑	↑	↑	↓	↑	1.13	2.24	+	-	-	NC
10	66	↑	↑	↑	↓	↑	2.35	1.92	+	-	-	NC
10	73	↑	↑	↑	↓	↑	2.21	2.00	+	-	-	NC
2	55	↑	↑	↑	↓	↑	2.65	2.82	+	-	-	NC

ELISA cut-off: 0.4.

## Discussion

The identification of *T. cruzi* by xenodiagnosis, hemoculture, microscopy or DNA based assays provides a definitive diagnosis for Chagas disease and can be routinely used to accurately diagnose the disease in the acute phase. However, during the indeterminate or chronic phase these tools are unreliable and a negative result may not mean lack of infection or parasitic cure of an infected patient, with serological methods being more informative.

Once a positive diagnosis has been confirmed, there are only two drugs available for treatment (Nfx and benznidazole) [38]. These drugs have significant side-effects [39] and their efficacy is controversial as there is currently no way to measure cure rates without decades of follow-up. There is consequently a lack of consensus on treatment duration throughout the community studying Chagas disease [40], and policy decisions and recommendations cannot be made. There is an urgent need for a reliable test that can determine cure in treated patients and measure treatment efficacy for a drug [41].

In our previous study we assessed cure in a cohort of adult patients treated with Nfx and followed up after three years by various diagnostic tools. Our results demonstrated the inadequacy of conventional serological tests for assessing cure in patients. In the cohort, 97.3% had results that could either indicate treatment failure or persistent humoral response despite having undergone treatment. On the other hand, the same samples all tested negative by conventional PCR and all but one tested negative by RT-PCR. These results reinforced the need for a robust and rapid test to assess treatment efficacy with certainty.

Research on the discovery and use of new antigens for diagnosis is on-going [42-44], but new diagnostic techniques such as proteomics [36] and flow cytometry [45] are also being investigated. In this study, we looked at the effect of Nfx in Chagas patients by using proteomics on samples from the same cohort of adults described in our previous study. We targeted our research on key biomarkers previously identified in our laboratories for diagnostic purpose [36] with a two-fold objective: assess whether specific biomarkers could be discriminative enough and help in defining cure in patients on the one hand and could be used as potential antigens for further development of new test of cure on the other hand.

Using SELDI, we successfully identified the majority of the key biomarkers discovered in our previous study [36] in the three test groups (healthy, infected and Nfx-treated follow-up samples), focusing on key biomarkers (mature human APOA1 (28.1 kDa); fragments of human APOA1 (24.7, 13.6 and 9.3 kDa); and a fragment of human FN1 (28.9 kDa). The pattern of these biomarkers between healthy and diseased samples was consistent with those seen in our previous study, i.e. all truncated fragments were up-regulated during disease compared to healthy controls whereas full-length APOA1 was downregulated during disease compared to healthy controls. These results, based on samples from Bolivian patients, confirm those previously seen with Venezuelan patient samples and demonstrate the reproducibility of SELDI technology across populations from South America [36]. Cluster plots showed that the levels of these biomarkers in follow-up samples of patients treated with Nfx returned to levels similar to those seen in healthy controls.

Seroconversion is currently the only surrogate marker of cure for Chagas disease. Given that all patients in this cohort were seropositive at the three year follow-up, we could not make any correlation with seroconversion.

Our five chosen biomarkers were shown to achieve up to 100% sensitivity and 98% specificity for CD, enabling the classification of follow-up samples as coming from healthy (or cured) or Chagas subjects. The results indicated that Nfx had no effect in 56.2% (n = 21/37) of the patients, one of whom was known to be still infected with *T. cruzi* (positive RT-PCR).

Nfx treatment length was found to be important, as nine out ten patients who pursued treatment for less than 40 days were still classified as Chagas disease patients. Of the 27 patients who followed Nfx treatment for more than 40 days, 25 of whom complied with the full 60 days of treatment, 15 (55.6%) were classified as healthy and would be considered as cured.

Taken together these results suggest that these biomarkers might be useful in assessing treatment efficacy in chagasic patients and could lead to the development of a test of cure. Moreover, their evaluation in animal models of Chagas disease could lead to improvements in the predictability of these models and their translation to the human disease during the drug discovery and development process.

Although very encouraging, it is important to point out that our study has limitations, and further studies will be needed to establish the real potential of these markers for their use as test of cure and assessment of treatment efficacy for Chagas disease. We were unable to correlate the biomarkers data with seroconversion, the only surrogate for Chagas disease, and had to rely on the discriminatory power of an algorithm using these markers. Since seroconversion in children is much quicker than in adults (1–2 years and decade(s) respectively), a follow-up study with sera samples from treated children would confirm a correlation between these biomarkers and seroconversion, and therefore cure. Indeed, access to samples of adults treated for Chagas disease with follow-up samples for decades is very limited. We also did not have serum samples corresponding to End of Treatment (EoT) for analysis. Indeed, being able to establish cure and perform counseling at the end of treatment would already be a major breakthrough, and would eliminate the need for a long follow-up. In addition, it may be possible to establish if down-regulation of key biomarkers is directly linked to the absence of cruzipain (cruzipain cleavage sites have been identified in the APOA1 and FN1, data not shown).

Finally, access to and analysis of samples originating from larger cohorts of patients treated with other drugs (e.g. Benznidazole) would be very useful and could further validate our findings.

## Conclusions

There is a lack of reliable tests for assessment of cure following treatment in chronic Chagas disease patients. In this study we identified a biomarker pattern strongly associated with CD. Using an algorithm, we showed that Nfx-treated patients that have the same biomarker profile/pattern as healthy controls were classified as cured. Further studies using samples from chagasic children would be useful to address the correlation between these biomarkers and seroconversion.

## Abbreviations

2008-CD+: 2008 Chagas positive; 2011-FU: 2011 follow-up; ACN: Acetonitrile; APOA1: Apolipoprotein A-I; CD: Chagas disease; CHCA: Alpha-cyano-4-hydroxycinnamic acid; ELISA: Enzyme-linked immunosorbent assay; FN1: Fibronectin; HC: Healthy control; HRP: Horseradish peroxidase; HUG: Geneva University Hospitals; IEF: Isoelectric focusing; IFA: Indirect immunofluorescence assay; IHA: Indirect hemaglutination assay; KLH: Keyhole limpet hemocyanin; MALDI: Matrix-assisted laser desorption ionization; Nfx: Nifurtimox; PAHO: Pan American Health Organization; PCR: Polymerase chain reaction; ROC: Receiver-operating characteristic curve; SELDI TOF: Surface-enhanced laser desorption ionization time-of-flight; TFA: Trifluoroacetic acid.

## Competing interests

The authors declare that they have no competing interests.

## Authors’ contributions

EC, YJ, FC, MN developed the study concept and design. CS, EC, YJ, QM, FC, MN carried out data acquisition. CS, YJ, QM, BW, FC, MN participated in the interpretation of the data. CS, EC, MN performed the statistical analysis. CS, EC, QM, BW, MN drafted or helped to draft the manuscript. All authors read and approved the final manuscript.

## Pre-publication history

The pre-publication history for this paper can be accessed here:

http://www.biomedcentral.com/1471-2334/14/302/prepub
